# Tissue oxygenation in patients with severe sepsis

**DOI:** 10.1186/cc13430

**Published:** 2014-03-17

**Authors:** A Oei, M Kok, A Karakus, H Endeman

**Affiliations:** 1 Academic Medical Center, Amsterdam, the Netherlands; 2 University Medical Center, Utrecht, the Netherlands; 3 Diakonessenhuis, Utrecht, the Netherlands; 4 Onze Lieve Vrouwen Gasthuis, Amsterdam, the Netherlands

## Introduction

The aim of this study was to determine the correlation between regional tissue oxygenation (SrO_2_) measured by near-infrared spectroscopy (NIRS), central venous oxygen saturation (SvO_2_) and levels of serum lactate (SL) in patients with severe sepsis.

## Methods

An observational pilot study was performed in an ICU of a medium-sized teaching hospital. Adult patients admitted with severe sepsis were included and three NIRS electrodes were attached: on the left side of the forehead (LF), the right side of the forehead (RF) and the right forearm (RA). SL and SvO_2 _measured in a jugular or subclavian central venous line sample were determined every 4 hours. Descriptive statistics were calculated. Pearson correlation coefficients (PCC) were calculated between SrO_2_, SvO_2 _and SL. Normal values for SrO_2 _were defined as the median ± 1 interquartile range in patients with SL <1.7 mmol/l. Differences between groups were calculated by Pearson chi-square tests. *P *< 0.05 was considered statistically significant.

## Results

Twenty-five patients (12 men) were included. Mean age was 68 years, mean APACHE score 31. The most frequent reasons for ICU admission were abdominal sepsis (*n *= 11) and pneumosepsis (*n *= 7). Statistically significant correlations were found between SvO_2 _and SrO_2_: PCC SrO_2 _LF, 0.46; PCC SrO_2 _RF, 0.50; PCC SrO_2 _RA, 0.21. Low, although statistically significant, correlations were found between SrO_2 _and SL: PCC SrO_2 _LF, -0.16; PCC SrO_2 _RF, -0.15; and PCC SrO_2 _RA, -0.20. Calculated normal values for SrO_2 _LF were 60 to 80%, for SrO_2 _RF were 60 to 76% and for SrO_2 _RA were 64 to 84%. An out-of-range SrO_2 _had a high positive predictive value (PPV) for an increased SL. The PPV for out-of-range SrO_2 _LF was 85% versus 58% for normal SrO_2 _LF (OR 4.27; 95% CI 2.09 to 8.72), the PPV for out-of-range SrO_2 _RF was 77% versus 60% for normal SrO_2 _RF (OR 2.32; 95% CI 1.25 to 4.31) and the PPV for out-of-range SrO_2 _RA 75% versus 60% for normal SrO_2 _RA (OR 1.94; 95% CI 0.95 to 4.00).

## Conclusion

This pilot study shows a statistically significant correlation between SvO_2 _and SrO_2 _in patients with severe sepsis. An out-of-range SrO_2 _LF or RF had a high predictive value for increased serum lactate. We therefore conclude that NIRS could have a role in goal-directed therapy of patients with severe sepsis.

